# Gender differences in patients with dizziness and unsteadiness regarding self-perceived disability, anxiety, depression, and its associations

**DOI:** 10.1186/1472-6815-12-2

**Published:** 2012-03-22

**Authors:** Annette Kurre, Dominik Straumann, Christel JAW van Gool, Thomas Gloor-Juzi, Caroline HG Bastiaenen

**Affiliations:** 1 Department of Rheumatology and Institute of Physical Medicine, University Hospital Zurich, Zurich, Switzerland; 2 Interdisciplinary Center for Vertigo & Balance Disorders, Departments of ENT, Neurology & Psychiatry, University Hospital Zurich, Zurich, Switzerland; 3 Maastricht University, school CAPHRI, Department of Epidemiology and Faculty of Health Medicine and Life Sciences, Maastricht, The Netherlands

## Abstract

**Background:**

It is known that anxiety and depression influence the level of disability experienced by persons with vertigo, dizziness or unsteadiness. Because higher prevalence rates of disabling dizziness have been found in women and some studies reported a higher level of psychiatric distress in female patients our primary aim was to explore whether women and men with vertigo, dizziness or unsteadiness differ regarding self-perceived disability, anxiety and depression. Secondly we planned to investigate the associations between disabling dizziness and anxiety and depression.

**Method:**

Patients were recruited from a tertiary centre for vertigo and balance disorders. Participants rated their global disability as mild, moderate or severe. They filled out the Dizziness Handicap Inventory and the two subscales of the Hospital Anxiety Depression Scale (HADS). The HADS was analysed 1) by calculating the median values, 2) by estimating the prevalence rates of abnormal anxiety/depression based on recommended cut-off criteria. Mann-Whitney *U*-tests, Chi-square statistics and odds ratios (OR) were calculated to compare the observations in both genders. Significance values were adjusted with respect to multiple comparisons.

**Results:**

Two-hundred and two patients (124 women) mean age (standard deviation) of 49.7 (13.5) years participated. Both genders did not differ significantly in the mean level of self-perceived disability, anxiety, depression and symptom severity. There was a tendency of a higher prevalence of abnormal anxiety and depression in men (23.7%; 28.9%) compared to women (14.5%; 15.3%). Patients with abnormal depression felt themselves 2.75 (95% CI: 1.31-5.78) times more severely disabled by dizziness and unsteadiness than patients without depression. In men the OR was 8.2 (2.35-28.4). In women chi-square statistic was not significant. The ORs (95% CI) of abnormal anxiety and severe disability were 4.2 (1.9-8.9) in the whole sample, 8.7 (2.5-30.3) in men, and not significant in women.

**Conclusions:**

In men with vertigo, dizziness or unsteadiness emotional distress and its association with self-perceived disability should not be underestimated. Longitudinal surveys with specific pre-defined co-variables of self-perceived disability, anxiety and depression are needed to clarify the influence of gender on disability, anxiety and depression in patients with vertigo, dizziness or unsteadiness.

## Background

The German National Telephone Health Interview Survey (GNT-HIS) in 2003 demonstrated a lifetime 29.5 prevalence of dizziness or vertigo in the adult population of Germany [1]. Vestibular vertigo accounted for a quarter of all reports of dizziness. Eighty percent of the affected individuals interrupted their work or daily activities as a result of the symptoms. Neuhauser et al. reported a strong association of vestibular vertigo and depression [1]. In 2006 Wiltink et al. estimated the prevalence of dizziness and anxiety in a representative sample of the German general population [2]. Sixteen percent of the participants reported symptoms of dizziness and 28.3 percent of these individuals had at least one anxiety disorder. Co-morbid anxiety was associated with increased health care use and disability. The mean level of disability rose with the number of dizziness and anxiety related symptoms [2]. In a primary care study performed in the United Kingdom a prevalence of dizziness of 23% was found [3]. About half of the individuals suffering from dizziness experienced some disability and 46% reported anxiety and/or avoidance of the movements and situations triggering dizziness and anxiety. The mean level of disability increased with the amount of co-morbid anxiety and avoidance [3]. More surveys report that psychological factors such as anxiety, depression, and autonomous arousal influence the level of disability experienced by patients with vertigo, dizziness or unsteadiness [1-8]. Furthermore these psychological factors seem to be risk factors for chronic dizziness and disability [8-11].

Prevalence rates of anxiety and depression in patients presenting with vertigo, dizziness or unsteadiness differ between previous surveys. For anxiety, values range from 11% to 40% [2,4,12-20]. For depressive mood, prevalence values range from 4% to 22% [4,6,12,14,15,17,18,20]. In comparison, in a general German population Hinz and Schwarz [21] estimated abnormal anxiety in 5.9%, and abnormal depression in 15.8% of the population. In this study the Hospital Anxiety and Depression Scale (HADS) was used [22]. The HADS is an often-used instrument for screening current states of anxiety (HADS-A) and depression (HADS-D), which we also applied in our survey.

In individuals with vertigo, dizziness and unsteadiness, gender differences have been described. The above mentioned GNT-HIS reported a higher lifetime prevalence of moderate or severe dizziness or vertigo in women (36%) compared to men (22%) [1]. Severe vestibular vertigo leading to interruption of daily or occupational activities was reported in 8.4% of women and 3.4% of men [1]. Comparable results are reported in other countries. Yardley et al. found that 12.7% of women reported handicapping dizziness compared to 8.4% of men [3]. Similarly, Piker and colleagues found that women with dizziness and unsteadiness tended to report higher levels of experienced handicap [4]. Beside higher prevalence rates of dizziness and higher levels of disability in women compared to men, gender differences were reported with respect to co-morbid anxiety and depression. Monzani et al. reported a prevalence of anxiety of 34% in patients with mixed vestibular disorders and interpreted this high rate to be the result of an overrepresentation of women (71% women) in their study-population [17]. Furthermore, they reported that women scored with a mean (standard deviation) score of 10.9 ± 5.9 points significantly higher on the HADS-A compared to men (5.86 ± 4.5). Piker et al. also reported an about two points (one point) higher mean score in HADS-A (HADS-D) in female patients with dizziness [4]. Gazzola and colleagues studied elderly people with chronic vestibular dysfunction and found that female gender was associated with the severity of depressive symptoms [23]. In contrast, Ketola et al. reported a higher but non significant prevalence rate of depression in male individuals suffering from vertigo (10% versus 9%) [6]. The previously mentioned survey of Hinz and Schwarz [21] in a general German population, also showed different prevalence rates between genders: abnormal anxiety was found in 7.4% of women and in 3.9% of men. Abnormal depression was found in 16.6% of women and 14.8% of men.

Gender differences regarding self-perceived disability, anxiety, depression, and its associations may be relevant for decision making within the diagnostic procedure and might call for a differentiation in treatment between female and male patients. Based on data previously collected to investigate the reliability and validity of the German version of the Dizziness Handicap Inventory (DHI-G) we planned this descriptive study. The primary objective of this secondary analysis was to explore gender differences in patients with dizziness and unsteadiness. Based on the above mentioned results of previous surveys, we were interested if we could find a trend that in women 1) the prevalence rates of self-rated severe disability, abnormal anxiety and abnormal depression, and 2) the mean scores of the applied questionnaires assessing self-perceived disability, anxiety and depression are higher than in men. Secondly we planned to explore the associations between self-perceived disability and anxiety and depression in our total study-population and in both genders separately. We furthermore wished to compare the prevalence of anxiety and depression in women and men of our study-population with the one of a German speaking general population.

## Methods

### Participants

Patients who had suffered for at least one month from vertigo, dizziness or unsteadiness were included in the study. The diagnosis of a vestibular disorder was made in the context of a previous or the present neuro-otological workup in our vertigo center. Further inclusion criteria were age between 18 and 75 years, the ability to walk, to independently manage about 50% of the daily tasks, and to understand and speak German. Exclusion criteria were dizziness or unsteadiness exclusively due to cardiopulmonary, musculoskeletal, neurological or psychic disorders [24]. These were identified by the clinicians (all neurologists) based on history and bedside testing, which also included a general medical assessment. If necessary, other specialists (internists, cardiologists, rheumatologists, psychiatrists etc.) were consulted.

### Procedure

In the period between July 2007 and July 2009, participants were recruited consecutively from the Interdisciplinary Center for Vertigo & Balance Disorders, Departments of ENT, Neurology & Psychiatry at the University Hospital Zurich. Patients were referred to the center primarily for diagnostic reasons. The diagnostic procedure consisted of a detailed clinical history, a complete neuro-otological bedside examination, laboratory tests, and MR imaging of the brain with special emphasis on brainstem, cerebellum and vestibulo-cochlear nerves. The distinction into the diagnostic subgroups was done in accordance with the guidelines of the DGN published in 2008 [25]. All patients who were referred to the vertigo center were asked to participate in this study. Clinical records of volunteers were screened by one of the authors (AK and TG) for matching the inclusion and exclusion criteria. Eligible patients, after giving written informed consent, received questionnaires assessing self-perceived disability, vertigo related symptoms, anxiety and depression as well as socio-demographic characteristics by regular mail. The procedure of collecting the questionnaires was supervised by two authors (TG and AK), who reminded patients to return the questionnaires in time and clarified missing or unclear responses [24,26]. The ethics committee of the Canton of Zurich approved the survey, which was the continuation of two studies on the reliability and validity of the DHI-G [24,26] and the Vertigo Symptom Scale (VSS).

### Measures

#### Disability: part 1: global level of disability

Patients were asked to rate their self-perceived disability caused by dizziness, vertigo or unsteadiness as mild, moderate or severe. To investigate the prevalence of self-rated severe disability, we created a dichotomous variable. Patients who rated their disability as mild or moderate composed one category. Patients who rated their disability as severe presented the second category of the dichotomous variable.

#### Disability: part 2: inventory of specific dizziness related disabilities

We applied the Dizziness Handicap Inventory (DHI), a 25-item questionnaire, which helps patients to rate dizziness-related physical and emotional impairments, activity limitations, and restrictions in participation [27]. A *yes *response gives a score of 4 points, *sometimes *2 points, and *no *0 points. The total score ranges from zero (no disability) to 100 (severe disability). Several validated translations and cross-cultural adaptations of the DHI exist [24,28-33]. The original questionnaire consists of three subscales [27] but the internal validity of the content domains could hardly be supported [26,28,34-36]. As suggested we only used the total scale. For the German version of the DHI (DHI-G) the values of the Cronbach's alpha and intraclass correlation coefficient (ICC) were 0.9 and 0.95 (95% confidence interval: 0.91-0.98). The estimated limits of agreement for the total scale were ± 12.4 points (95% CI: 9.0 to 15.8 points) [24].

#### Symptom severity: vertigo and somatic anxiety related symptoms

These aspects were assessed with the two subscales of the Vertigo Symptom Scale (VSS) [37]. The VSS-VER assesses 'vertigo and related symptoms', the VSS-AA 'somatic anxiety and autonomic arousal'. Respondents indicate how often they suffered during the last 12 month from 22 symptoms: *never *(0 points), *1*-*3 times a year *(1 point), *4-12 times a year *(2 points), *more than once a month *(3 points), *more than once a week *(4 points). The two subscales demonstrated good internal consistencies (Cronbach's alpha coefficients: VSS-VER 0.86; VSS-AA 0.86) and reliability (ICCs: VSS-VER 0.92; VSS-AA 0.91) [Gloor-Juzi et al. unpublished]. Furthermore Tschan et al. (2008) supported the original two-factor structure as obtained by Yardley and co-workers [38]. The 19 item VSS-VER discriminated sufficiently between patients and healthy controls. The 15 item VSS-AA discriminated moderately between somatoform dizziness and dizziness caused by neuro-otological disorders [38].

#### Anxiety and depression

The 14-item Hospital Anxiety and Depression Scale (HADS) was used to assesses non-somatic symptoms of anxiety (HADS-A) and depression (HADS-D) [39]. Each item is rated with 0-3 points. Scores on the two subscales range from zero (no sign of anxiety or depression) to 21 (maximum level of anxiety or depression). Factor analyses of the German version of the HADS confirmed the original two-factor structure of the HADS [21,22]. The internal consistencies were shown to be good (HADS-A: 0.93; HADS-D: 0.90) [22]. Across several language versions retest reliability showed a high correlation (r > 0.80) after up to 2 weeks [40]. The validity of the HADS has been demonstrated across a number of patient groups [40]. A good positive predictive value (85%) was shown among otolaryngology patients [41]. Zigmond and Snaith recommended a cut-off score of ≥ 8 for 'possible' and ≥ 11 for 'probable' anxiety or depression [39]. For the German version of the HADS the suggested cut-off criteria for 'abnormal anxiety' is ≥ 11 and for 'abnormal depression' ≥ 9 [22].

### Statistical analyses

For the purpose of this survey to explore gender differences and to estimate prevalence rates, neither a sample size calculation nor a specific data collection was performed. All analyses are based on previously collected data.

Baseline characteristics of the study population such as the absolute and relative frequencies of the total population, women and men in the different groups of interest (groups of diagnoses, illness duration etc.) were described. Pearson's chi-square statistics were calculated to estimate if the distribution of both genders was different between these categorical variables.

The mean values and standard deviations (SD) respectively medians and interquartil ranges (IQR) of the different questionnaire-scores were calculated. To assess if both genders differ in the mean respectively median values of the DHI-G (disability), VSS-VER and VSS-AA (symptom-severity), HADS-A (anxiety), and HADS-D (depression) Student *T*-tests or Mann-Whitney-*U *tests were performed. We planned to apply an alpha value of 0.01 [dividing the initial alpha of 0.05 by the number of comparisons (five)].

In order to investigate whether both genders differ in the prevalence of self-rated severe disability, abnormal anxiety and depression, Mantel Haenszel chi-square statistics were performed and the common odds ratios (OR) with the 95% confidence intervals (CI) were calculated. The alpha used is 0.017 as the initial alpha of 0.05 was divided by the number of comparisons (three).

Multiple Kruskal-Wallis tests were performed to assess if the median values of the DHI-G, HADS-A and HADS-D differ significantly in the different categories of diagnostic groups, illness duration, employment status and living condition. Multiple Pearson chi-square statistics were done to investigate the associations between the above mentioned categorical variables and the dichotomous variables disability, anxiety and depression.

To describe the associations of self-perceived disability, anxiety and depression we performed several statistics: 1) Mantel Haenszel chi-square statistics and common OR's were calculated to analyse the associations between the dichotomous variables disability and anxiety and depression. The alpha used is 0.0125 (0.05 divided by 4). 2) We compared the median values of the DHI-G, HADS-A, and HADS-D in the subgroups with different levels of disability, anxiety and depression. The corrected alpha value is 0.006 (0.05 divided by 8). 3) We estimated the associations between the DHI-G and HADS by calculating Spearman correlation coefficients. Coefficients of 0.26 - 0.50 were considered to indicate fair associations, 0.51 - 0.75 moderate and ≥ 0.76 strong associations [42]. Partial correlations were calculated to estimate the association of self-perceived disability (DHI-G) and emotional distress (HADS) by controlling for the effect of symptom severity (VSS) on both variables.

The analyses were computed using the PASW version 18.0 software.

## Results

**Figure 1:**
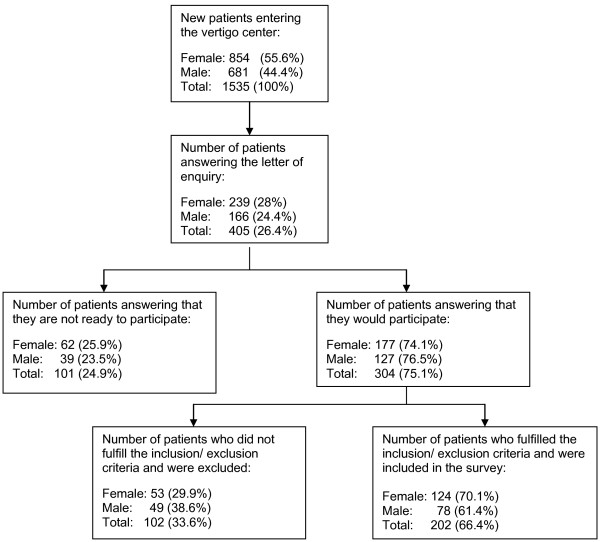
**Recruitment of the patients**.

During the time of recruitment 1 535 new patients entered our vertigo center for diagnostic reasons. Four hundred and five (26.4%) of the patients answered the letter of enquiry whether they are willing to participate in the survey. One hundred and one (25%) individuals were not, 304 (75%) were ready to participate. Two hundred and two patients (66.4%) could be included in the survey because they fulfilled the inclusion and not the exclusion criteria (Figure 1).

### Baseline characteristics of the study population

Of the 202 participants 124 (61.4%) were female, 78 (38.9%) male. Both genders did not differ significantly in mean age (Table 1). Two male patients did not completely fill out the HADS. Therefore only 200 valid data sets could be analysed regarding anxiety and depression.

**Table 1:** Baseline characteristics of the study population (n = 202)

	Total sample	Female	Male	gender comparisons
Total sample (n [%])	202 (100)	124 (61.4)	78 (38.6)	

Age (mean [SD])	49.7 (13.5)	50.2 (13.2)	48.9 (14.1)	n.s.^1^

Groups of diagnoses (n [%])				

UPVD	77 (38.1)	46 (37.1)	31 (39.7)	n.s.^2^
	
BPVD	18 (8.9)	9 (7.3)	9 (11.5)	
	
Psychophysic dizziness	20 (9.9)	14 (11.3)	6 (7.7)	
	
Vestibular migraine	27 (13.4)	19 (15.3)	8 (10.3)	
	
Multiple vestibular disorders	16 (7.9)	11 (8.9)	5 (6.4)	
	
CVD	19 (9.4)	10 (8.1)	9 (11.5)	
	
Multifactorial dizziness	25 (12.4)	15 (12.1)	10 (12.8)	

Illness duration (n [%])				

> 1 mo, < 6 mo	58 (28.7)	37 (29.8)	21 (26.9)	n.s.^2^
	
> 6 mo, < 12 mo	27 (13.4)	15 (12.1)	12 (15.4)	
	
> 12 mo	117 (57.9)	72 (58.1)	45 (57.7)	

Employment status (n [%])				

not employed	61 (30.2)	43 (34.7)	18 (23.1)	p^2 ^= 0.000
	
< 50%	23 (11.4)	17 (13.7)	6 (7.7)	
	
50 - 79%	25 (12.4)	23 (18.5)	2 (2.6)	
	
> 80%	93 (46.0)	41 (33.1)	52 (66.7)	

Living condition (n [%])				

alone	39 (19.3)	20 (16.1)	19 (24.4)	n.s.^2^
	
with adult partner	97 (48.0)	61 (49.2)	36 (46.2)	
	
with adult partner and childs	57 (28.2)	34 (27.4)	23 (29.5)	
	
with childs	9 (4.5)	9 (7.3)	0	
	
DHI-G (mean [SD])	44.6 (22.2)	45.8 (21.7)	42.6 (23.0)	n.s.^1^

HADS-A* (median [IQR])	6.0 (6.0)	6.0 (6.0)	6.0 (7.0)	n.s.^3^

HADS-D* (median [IQR])	4.0 (5.0)	4.0 (5.0)	4.0 (7.0)	n.s.^3^

VSS-VER (median [IQR])	17.0 (18.0)	17.0 (18.0)	14.5 (16.0)	n.s.^3^

VSS-AA (median [IQR])	17.0 (15.0)	18.0 (15.0)	14.0 (12.75)	n.s.^3^


* n = 200

^1 ^= Student *t*-test

^2 ^= Pearson chi-square statistic

^3 ^= Mann-Whitney U

*BPVD *indicates bilateral peripheral vestibular dysfunction, *CVD *central vestibular dysfunction, *HADS *Hospital Anxiety and Depression Scale, *HADS-A *anxiety subscale of the HADS (score: 0-21), *HADS-D *depression subscale of the HADS (score: 0-21), *mo *month, *n.s. *not significant, *UPVD *unilateral peripheral vestibular dysfunction, *VSS *Vertigo Symptom Scale, *VSS-VER *subscale measuring 'vertigo and related symptoms' (Score: 0-76), *VSS-AA *subscale measuring 'somatic anxiety and autonomic arousal' (Score: 0-60).

Of all of the participants 80.2% performed their daily activities without personal assistance, 13.5% needed personal assistance less than once a week, and 6.5% at least weekly. The number (%) of patients in the different groups of diagnoses, illness duration, employment status, and living condition are reported in Table 1. The distribution of women and men did not differ significantly in the diverse groups of diagnoses, illness duration, and living condition. A more detailed composition of the diagnostic groups can be seen in the Additional file 1: Table S1. In Additional file 2: Table S2, the distribution of co-morbidities in different body-structures is reported.

### Comparison of the mean level of self-perceived disability, anxiety and depression in both genders

In female and male patients the differences of the mean respectively median values of the DHI-G (self-perceived disability), VSS-VER, VSS-AA (symptom severity), HADS-A, and HADS-D (anxiety and depression) were not significant (Table 1).

### Comparison of the prevalence rates of self-rated severe disability, abnormal anxiety and depression in both genders

There was a tendency of a higher prevalence of severe disability in women (25%) compared to men (19.2%), and a higher prevalence of abnormal anxiety and depression in men (23.7%; 28.9%) compared to women (14.5% and 15.3%) (Table 2). Men with vertigo, dizziness or unsteadiness were 2.26 (1.1- 4.5) times more likely to be depressive compared to women. Taking into account the multiple testing and the correction of the significance value this result was not any more significant.

**Table 2:** Frequencies of female and male participants in subgroups of disability, anxiety and depression

Variables	Categories	Female (n = 124) (n [%])	Male (n = 76) (n [%])	Chi-square statistics
Severe disability^a^	present	31 (25.0)	15 (19.2)	*x*^2 ^(1) = 0.467;
	
	not present	93 (74.9)	63 (80.8)	p = 0.494

Abnormal anxiety^b^	present	18 (14.5)	18 (23.7)	*x*^2 ^(1) = 2.088;
	
	not present	106 (85.5)	58 (76.3)	p = 0.148

Abnormal depression^c^	present	19 (15.3)	22 (28.9)	*x*^2 ^(1) = 4.541;
	
	not present	105 (84.7)	54 (71.0)	p = 0.033

^a ^Subjects who rated their disability as mild or moderate were put together in the category 'severe disability not present

^b ^Anxiety was assessed with the anxiety subscale of the Hospital Anxiety and Depression Scale (HADS). Subjects with scores of ≥ 11 points were found to have abnormal anxiety [40].

^c ^Depression was assessed with the depression subscale of the HADS. Subjects with scores ≥ 9 points in Chi-square statistic was calculated with the Mantel-Haenszel test. HADS-D were found to have abnormal depression [40].

An adjustment of the significance value was calculated: alpha/n; n = number of comparisons; alpha = 0.05: corrected significance level: 0.05/3 = 0.017

### Associations between the groups of diagnoses, illness duration, employment status and living condition and disability, anxiety and depression

The median values of the DHI-G, HADS-A and HADS-D did not significantly differ in the subgroups of these categorical variables. Pearson chi-square statistics did not result in significant associations between the above mentioned categorical variables and the dichotomous variables of disability, anxiety and depression.

### Associations between self-perceived disability and anxiety and depression

In the whole study-population the association of the dichotomous variables anxiety and disability was significant: Mantel-Haenszel *x*^2 ^(1) = 12.86, p = 0.000; OR = 4.2 (1.9 - 8.9). The same was the case between the association of depression and disability: Mantel-Haenszel *x*^2 ^(1) = 6.38, p = 0.012; OR = 2.8 (1.3 - 5.8). Analysing these associations in both genders resulted in significant Mantel-Haenszel chi-square statistics only in men: Men with dizziness and anxiety felt themselves 8.7 (2.5 - 30.3) times more severe disabled than men with dizziness without anxiety. Men with dizziness and depression felt themselves 8.2 (2.4 - 28.4) times more severe disabled than men with dizziness without depression (Table 3). These results were significant with values below the corrected significance level of p = 0.0125.

**Table 3:** Associations between the prevalence rates of disability and anxiety and depression in both genders

Gender	Severe disability^a^	Abnormal anxiety^b^		Severe disability^a^	Abnormal depression^c^	
		present (n [%])	not present (n [%])		present (n [%])	not present (n [%])
F	present	8 (6.5)	23 (18.5)	present	6 (4.8)	25 (20.2)
	
	not present	10 (8.1)	83 (66.9)	not present	13 (10.5)	80 (64.5)

		*x*^2 ^(1) = 3.09, p = 0.079;			*x*^2 ^(1) = 0.185, p = 0.667	

M	present	9 (11.8)	6 (7.9)	present	10 (13.2)	5 (6.5)
	
	not present	9 (11.8)	52 (68.4)	not present	12 (15.8)	49 (64.5)

		*x*^2 ^(1) = 11.1, p = 0.001; OR: 8.7 (2.5 - 30.3)			*x*^2 ^(1) = 10.60, p = 0.001; OR: 8.2 (2.4 - 28.4)	

^a ^Subjects who rated their disability as mild or moderate were put together in the category 'severe disability not present'

^b ^Anxiety was assessed with the anxiety subscale of the Hospital Anxiety and Depression Scale (HADS). Subjects with scores of ≥ 11 points were found to have abnormal anxiety [40].

^c ^Depression was assessed with the depression subscale of the HADS. Subjects with scores ≥ 9 points in HADS-D were found to have abnormal depression [40].

*F *indicates female (n = 124), *M *male patients (n = 76), *OR *odds ratio.

Chi-square statistic was calculated with the Mantel-Haenszel test.

An adjustment of the significance value was calculated: alpha/n; n = number of comparisons; alpha = 0.05: corrected significance level: 0.05/4 = 0.0125

Patients with mild, moderate or severe disability did not differ significantly in mean age, but in the median values of HADS-A and HADS-D. In men with self-rated severe disability the median values of HADS-A and HADS-D were significantly higher than in women and exceeded with 12 points the cut-off criteria for anxiety and depression (Table 4).

**Table 4:** The level of anxiety, depression, and disability in both genders with different health conditions

			N (%)	HADS-A Median (IQR)	Statistic	HADS-D Median (IQR)	Statistic
	Disability	mild	34 (27.4)	4.5 (5.0)	p^1 ^= 0.025	2.0 (2.5)	p^1 ^= 0.001
					
F (124)		moderate	59 (47.5)	6.0 (6.0)		4.0 (5.0)	
					
		severe	31 (25.0)	7.0 (6.0)		6.0 (3.0)	

		mild	22 (28.2)	3.0 (4.5)	p^1 ^= 0.007	1.0 (6.0)	p^1 ^= 0.000
					
M (76)		moderate	41 (52.6)	6.0 (5.0)		4.0 (5.0)	
					
		severe	15 (19.2)	12.0 (11.0)		12.0 (9.0)	

				DHI-G Mean (SD)			

F (124)	Abnormal anxiety	yes	18 (14.5)	60.1 (22.0)	p^2 ^= 0.002		
				
		no	106 (85.5)	43.4 (20.8)			

M (76)		yes	18 (23.7)	63.6 (20.6)	p^2 ^= 0.000		
				
		no	58 (76.3)	36.0 (20.2)			

				DHI-G Mean (SD)			

F (124)	Abnormal depression	yes	19 (15.3)	66.5 (16.0)	p^2 ^= 0.000		
				
		no	105 (84.7)	42.1 (20.6)			

M (76)		yes	22 (28.9)	65.7 (16.2)	p^2 ^= 0.000		
				
		no	54 (71.0)	33.1 (18.7)			

^1 ^= Kruskal-Wallis test

^2 ^= Student *t*-test

*DHI-G *indicates Dizziness Handicap Inventory - German version (Score: 0 - 100), *F *female, *HADS *Hospital Anxiety and Depression Scale, *HADS-A *anxiety subscale (Score: 0 - 21), *HADS-D *depression subscale of the *HADS *(Score: 0 - 21), *IQR *interquartil range, *M *male, *SD *standard deviation

An adjustment of the significance value was calculated: alpha/n; n = number of comparisons; alpha = 0.05: corrected significance level: 0.05/8 = 0.00626.

Table 5 shows the associations between the DHI-G and the HADS respectively HADS-D. In both genders the Spearman correlation coefficients were of moderate size, but the values were higher in men. The partial correlations between the DHI-G and the HADS respectively the HADS-D controlling for the effect of symptom severity as assessed with the VSS remained of moderate size only in men.

**Table 5:** Associations between the Dizziness Handicap Inventory and the Hospital Anxiety and Depression Scale

Sample	n (%)	HADS		HADS-D		HADS-A	
		**Corr**.	**Partial Corr**.	**Corr**.	**Partial Corr**.	**Corr**	**partial Corr**.

All	200	0.60**	0.44**	0.66**	0.50**	0.45**	0.30**

Female	124 (62)	0.53**	0.38**	0.63**	0.47**	0.35**	0.22**

Male	76 (38)	0.72**	0.53**	0.74**	0.57**	0.59**	0.41**

** significant at the 0.01 level (1-tailed)

*Corr *indicates the Spearman correlation coefficients, *HADS-A *anxiety subscale of the HADS, *HADS-D *depression subscale of the HADS, *partial corr *partial correlation: a measure of the association between the DHI and HADS controlling for the effect of symptom severity (Vertigo Symptom Scale, Dizziness Index) on the DHI and HADS.

### Comparison of the prevalence rates of abnormal anxiety/depression with reference values

In Table 6 it can be seen that in the male participants of our study-population the prevalence of abnormal anxiety and depression was 6-times respectively 2-times higher compared to the male reference population [21]. In women only the prevalence of abnormal anxiety was about 2-times higher than the one of the female reference population.

**Table 6:** Prevalence rates of anxiety and depression in our sample compared to a reference population

	Anxiety^a^			Depression^b^		
**Sample**	**Sample %**	**Reference^c ^%**	**Quotient Sample/Ref**.	**Sample %**	**Reference^c ^%**	**Quotient Sample/Ref**.

Total	18.0	5.9	3.1	20.5	15.8	1.3

Female	14.5	7.4	1.9	15.3	16.6	0.9

Male	23.7	3.9	6.1	28.9	14.8	2.0

^a ^Anxiety was assessed with the anxiety subscale of the Hospital Anxiety and Depression Scale (HADS). Subjects with scores of ≥ 11 points were found to have anxiety [40].

^b ^Depression was assessed with the depression subscale of the HADS. Subjects with scores of ≥ 9 points were found to have depression [40].

^c ^Reference. Hinz and Schwarz (2000) estimated the prevalence rates of anxiety and depression in a representative sample of the adult population of Germany.

## Discussion

In 202 patients (61.4% female) with vertigo, dizziness or unsteadiness we investigated gender differences regarding self-perceived disability, anxiety, depression, and its associations. Both genders did not differ significantly in the mean level of self-perceived disability, anxiety, depression, and symptom severity. In men with vertigo, dizziness or unsteadiness the prevalence rates of abnormal anxiety and especially abnormal depression were higher than in women. With respect to the corrected significance value the association between gender and depression was not significant anymore. Our results showed that the associations of abnormal depression and anxiety with self-rated severe disability were significantly stronger in male than in female patients. In comparison to reference values of a general population of Germany [21] especially in men the prevalence rates of abnormal anxiety and depression were higher (6-fold respectively 2-fold).

As mentioned in the introduction in study-populations of subjects with dizziness, vertigo or unsteadiness the prevalence rates of anxiety and depression vary [2,4,6,12-20]. Diverse patient characteristics for example age, diagnoses, extent of disability, cultural and social aspects and different assessments of anxiety and depression might explain this. The values estimated by us are with 18% at the lower end of previously reported prevalence rates for anxiety and with 20.5% at the upper end of reported prevalence rates of depression. To our knowledge, none of the prior studies explicitly analysed gender differences in the prevalence of anxiety and depression in subjects with vertigo, dizziness or unsteadiness. However, several surveys that estimated the prevalence rates of anxiety and depression in German speaking general populations [21,40,43-49] reported higher prevalence rates in women compared to men [21,44,46-49]. This raises the questions why we found a different result. One explanation for our conflicting result might be that the higher prevalence rates in women before the onset of dizziness and unsteadiness were picked up by men with the onset of disabling dizziness. Possibly, vertigo or dizziness and associated problems may be greater risk factors for the development of anxiety and depression in men than in women. Because anxiety is described as one cause of depression respectively the development of co-morbid anxiety and depression [45,50] this may explain why in our sample the prevalence rates of depression (28.9% in men; 15.3% in women) and anxiety (23.7% in men; 14.5% in women) have been of almost the same size when compared within the same gender. About 70% of the patients included in our study suffered ≥ 6 month from vertigo, dizziness or unsteadiness. This might have been the time in which co-morbid anxiety and depression developed.

Different estimations of the level of emotional distress in study-populations composed of individuals with vertigo, dizziness or unsteadiness have been reported. Again the differences may be explained by the diverse size and composition of study-populations, the chosen assessment tools and statistical analyses. We analysed the surveys which applied the HADS [4,12,15,17,18]. Reported median respectively mean values of the HADS-A (HADS-D) range from 5 (4) in study-participants with peripheral vestibular disorders in Sweden [12] to 12.6 (6.7) in participants with mixed vestibular disorders in Italy [17]. We estimated in both genders median values of 4 in HADS-D which equals the reference values of Hinz and Schwarz [21]. The estimated median values of 6 points in HADS-A lie two points above the reference values. This may support the hypothesis that the occurrence of vertigo, dizziness or unsteadiness is primarily associated with anxious feelings.

In our survey in the whole study population, age, the different groups of diagnoses, illness duration, employment status, and living condition were not related to anxiety, depression and self-perceived disability. The strongest associations were found between self-perceived disability and anxiety and depression. This association was described in numerous previous studies as mentioned in the introduction [1-8]. We estimated in both genders not only the Spearman correlation coefficients between the DHI-G and HADS but also the associations of the dichotomous variables assessing anxiety respectively depression and self-rated disability. Of the men who rated their disability caused by vertigo, dizziness or unsteadiness as severe 60% had abnormal anxiety and 66.7% suffered from abnormal depression. In women about 26% of the ones with severe disability had anxiety and 19% depression. Especially in male patients the simple self-rating of the severity of disability as mild, moderate or severe might indicate that a patient should be screened for anxiety and depression if he rates the perceived disability as severe.

Our study has some limitations. The HADS is a useful screening instrument which covers relevant emotional distress in subjects with vertigo, dizziness or unsteadiness. But as a screening instrument the HADS gives only limited information on mental and behavioural disorders. The anxiety subscale of the HADS is primarily composed of statements relevant to generalised anxiety [51,52]. One item assesses the prevalence of panic-attacks. This item will not be sufficient to assess the prevalence of panic-disorders which represented the largest group of comorbid anxiety followed by generalized anxiety disorders and social phobia according to Wiltink et al. [2]. The items of the depression subscale assess simply symptoms of anhedonia (loss of pleasure response). These symptoms belong according to DSM-II-R and ICD-10 to the leading symptoms of the depressive episode, which constitutes only one of several mood disorders [51,52]. The fact that the HADS only assesses non-somatic symptoms of anxiety and depression may have led to an underestimation of anxiety and depression and possible gender differences, which are described in somatic symptom, may have been undetected. A further weakness of the HADS lies in the different recommended cut-off scores. Beside the suggested cut-offs of the original as well as the German version of the HADS [22,39] Bjelland and colleagues reported in their review that in most studies caseness was defined by a score of ≥ 8 [53]. A replication of our analyses based on these cut-offs resulted in an increase and equalization of the prevalence rates of anxiety in women and men (34.7%, 32.9%) and a major difference of the prevalence rates of depression in women (17.7%) and men (32.9%). The associations of the categorical variables 'depression' and 'self-perceived severe disability' remained significant only in men. Despite these disadvantages the HADS helps to identify patients with anxiety and depression that should become more specifically assessed and treated by specialists.

In our study-population, female and male patients neither differed significantly in the mean level of self-perceived disability as assessed with the DHI-G, nor in the proportion of both genders in the categories of mild, moderate or severe self-rated disability. Because we found stronger associations between anxiety respectively depression and disability in men compared to women this provokes the question which factors contributed to the level of disability in female patients. In each case we investigated only a small number of possible relevant factors associated with anxiety, depression and disability in patients with vertigo, dizziness or unsteadiness. One of these factors might be the type of vestibular pathology. Some surveys comparing patient groups with different vestibular disorders showed that patients with vestibular migraine and Menière's disease experienced more anxiety, depression and disability than patients with vestibular neuritis or benign paroxysmal positional vertigo [5,54,55]. In our survey the subgroup of patients suffering from vestibular migraine (n = 27) did not significantly differ from the other groups in their mean level of anxiety, depression and disability. Further surveys in patients with dizziness and unsteadiness investigated the effect of general co-factors that are known to be associated with emotional distress. Such factors are co-morbidities (e.g. migraine, coronary heart disease, chronic pulmonary disease, cerebrovascular disease, menopause) [1,2,10,11,23], health behaviour (e.g. smoking, drinking) [1,2,10], social status (e.g. income, partnership, employment, family, friends) [1,2,10,15,56], education [1,2,10,57], personality [10,18,58], and the kind of perceptions [3,7,8,10,13,15,19,56,57,59,60], cognitions [7,8,13,15,57-60] or coping strategies/illness behaviour [3,4,7,8,11,13,19,56,58,59]. Because of our retrospective analyses we did neither assess specifically these general nor gender specific risk factors of mental disorders. Risk factors for depression that disproportionately affect women include: low socio-economic status, low level of education, housewife, married, mother, single mother, unemployed, poor social support, responsible for family members in need of care, low mastery, violation in childhood, demanding life events, lower self-esteem, self-incrimination, rumination, pregnancy, birth [61-63]. Risk factors which might primarily affect men are: living alone, being divorced, problems at work, reduced bonus payment, retirement, chronic disease, limited perception of need, difficulties in help seeking [61-63].

Our study results can only be generalized with caution. Analyses are based on data primarily collected for a reliability and validity study of the DHI-G. For this study a hypotheses based power calculation was not performed. We recruited patients consecutively with the result of a higher proportion of women participating. This might have affected the investigation of gender differences. Only 26% of the new patients entering our centre answered the letter of enquiry. We do not know whether the patients willing to participate were comparable to the patients who did not answer.

We did not perform a multivariate analysis first of all because of our cross-sectional design. As such the results of our survey are purely descriptive and do not give insight into causal inferences.

This leads to suggestions for future research. Possible risk factors for the development of psychiatric distress and severe disabling dizziness should systematically be investigated in longitudinal studies. Some studies have started to investigate these relationships [5,8,11,13,56-60]. Because perceptions, emotions, cognitions and behaviour may change during the course of disease [13] the assessment of these aspects should be done in several time intervals to find out which symptoms and signs and what time points are most relevant. Because in our study-population the median values of the DHI-G and HADS did not differ in both gender, however, gender differences could be shown between women and men with extreme health problems, this should be analysed in more detail in future longitudinal studies. Age dependent gender differences in the prevalence of specific mood disorders, gender differences in the risk factors, symptoms and signs of mood disorders, gender bias in the diagnostic procedures are challenges which also have to be considered in future research in this field.

## Conclusions

Contrary to our expectations we could not find that women with vertigo, dizziness and unsteadiness suffer more from self-perceived disability, anxiety and depression than men. In male patients with vertigo, dizziness or unsteadiness there was a tendency of a higher prevalence of abnormal depression and a stronger association between anxiety and depression and self-perceived disability. Further surveys are needed to investigate gender specific differences regarding the development, the characteristics and the associations of self-perceived disability, anxiety and depression in patients with vertigo, dizziness or unsteadiness.

## Competing interests

The authors declare that they have no competing interests.

## Authors' contributions

AK: contributed to the design of the study, the acquisition and interpretation of the data, conducted the statistical analysis, and wrote the manuscript. DS: co-initiated the study, contributed to the interpretation of data, and revised the article critically for its content. CJAWvG: attributed to the design of the study, contributed to the interpretation of data and revised the article critically for its content. TG-J: contributed to the design of the study, the acquisition of data, and revised the article critically for its content. CHGB: contributed to the analysis and interpretation of the data, revised the article critically for its content, and gave the final approval of the version to be published. All authors read and approved the final manuscript.

## Pre-publication history

The pre-publication history for this paper can be accessed here:

http://www.biomedcentral.com/1472-6815/12/2/prepub

## Supplementary Material

Additional file 1 **Table S1 Number of female and male patients with specific diagnoses**. Click here for file

Additional file 2 **Table S2 Co-morbidities**. Click here for file
