# Supplementary material for: Gender differences in patients with dizziness and unsteadiness regarding self-perceived disability, anxiety, depression, and its associations
Source: BMC Ear Nose Throat Disord. 2012 Mar 22;12:2. doi: 10.1186/1472-6815-12-2 (PMC3352112; doi:10.1186/1472-6815-12-2)
Supplement: Additional file 1 — Table S1 Number of female and male patients with specific diagnoses. [file 1472-6815-12-2-S1.DOC]

**Additional Table 1** Number of female and male patients with specific diagnoses

| Diagnoses | Total sample  n (%) | Female  n (%) | Male  n (%) |
| --- | --- | --- | --- |
| Unilateral BPPV^1^ | 27 (13.4) | 20 (16.1) | 7 ( 9) |
| Unilateral Morbus Menière^1^ | 27 (13.4) | 14 (11.3) | 13 (16.7) |
| Unilateral Neuritis vestibularis^1^ | 4 ( 2.0) | 1 ( 0.8) | 3 ( 3.8) |
| UPVD^1^ | 19 ( 9.4) | 11 ( 8.9) | 8 (10.3) |
| Bilateral BPPV^2^ | 3 ( 1.5) | 3 ( 2.4) | 0 |
| Bilateral Morbus Menière^2^ | 1 ( 0.5) | 0 | 1 ( 1.3) |
| Bilateral Neuritis vestibularis^2^ | 4 ( 2.0) | 1 ( 0.8) | 3 ( 3.8) |
| BPVD^2^ | 10 ( 5.0) | 5 ( 4.0) | 5 ( 6.4) |
| Psychophysic dizziness | 20 ( 9.9) | 14 (11.3) | 6 ( 7.7) |
| Vestibular migraine | 27 (13.4) | 19 (15.3) | 8 (10.3) |
| CVD | 19 ( 9.4) | 10 ( 8.1) | 9 (11.5) |
| Multiple vestibular disorders | 16 ( 7.9) | 11 ( 8.9) | 5 ( 6.4) |
| Multifactorial dizziness | 25 (12.4) | 15 (12.1) | 10 (12.8) |
| total | 202 (100) | 124 (100) | 78 (100) |

^1^ indicates the diagnoses included in the diagnostic group ´unilateral peripheral vestibular

Disorder` (UPVD)

^2^ indicates the diagnoses included in the diagnostic group ´bilateral peripheral vestibular

Disorder` (BPVD)
