# Supplementary material for: Gender differences in patients with dizziness and unsteadiness regarding self-perceived disability, anxiety, depression, and its associations
Source: BMC Ear Nose Throat Disord. 2012 Mar 22;12:2. doi: 10.1186/1472-6815-12-2 (PMC3352112; doi:10.1186/1472-6815-12-2)
Supplement: Additional file 2 — Table S2 Co-morbidities. [file 1472-6815-12-2-S2.DOC]

**Additional Table 2** Co-morbidities

| Co-morbidities | Total sample  n (%) | Female  n (%) | Male  n (%) |
| --- | --- | --- | --- |
| No documented secondary diagnosis | 98 (48.5) | 64 (51.6) | 34 (43.6) |
| Musculosceletal disorder | 19 ( 9.4) | 11 ( 8.9) | 8 (10.3) |
| Cardiopulmonal disorder | 7 ( 3.5) | 3 ( 2.4) | 4 ( 5.1) |
| Disorder of the viscera | 8 ( 4.0) | 4 ( 3.2) | 4 ( 5.1) |
| Sensory/ perceptive dysfunction | 16 ( 7.9) | 10 ( 8.1) | 6 ( 7.7) |
| Pain | 9 ( 4.5) | 6 ( 4.8) | 3 ( 3.8) |
| Neurological disorder/  Neuromotoric dysfunction | 18 ( 8.9) | 8 ( 6.5) | 10 (12.8) |
| Multiple disorders | 27 (13.4) | 18 (14.5) | 9 (11.5) |
| total | 202 (100) | 124 (100) | 78 (100) |
